# A Cognitive Dual Task Alters Dynamic Tibiofemoral Movements During Jump-Landing Assessments in Healthy Participants

**DOI:** 10.1177/23259671251340996

**Published:** 2025-05-28

**Authors:** Tom Vendrig, Michèle N.J. Keizer, Reinoud W. Brouwer, Han Houdijk

**Affiliations:** † Department of Human Movement Sciences, University Medical Center Groningen, University of Groningen, Groningen, the Netherlands; ‡ Department of Orthopedic Surgery, Martini Hospital, Groningen, the Netherlands; Investigation performed at University Medical Center Groningen, University of Groningen, Groningen, the Netherlands

**Keywords:** anterior cruciate ligament, tibiofemoral movements, anterior tibial translation, internal tibial rotation, cognitive load, dual task, injury risk, jumping assessments

## Abstract

**Background:**

Many sports situations impose high cognitive demands on athletes, requiring them to divide their attention across multiple tasks and leading to landing mechanics associated with an increased anterior cruciate ligament (ACL) load and injury risk.

**Purpose:**

To investigate the influence of a cognitive dual task on dynamic tibiofemoral movements (ie, dynamic anterior tibial translation [ATTd] and dynamic internal tibial rotation [ITRd]) during jump-landing assessments in healthy participants.

**Study Design:**

Controlled laboratory study.

**Methods:**

A total of 20 healthy participants performed the side hop for distance, single-leg hop for distance, and triple hop for distance. Each assessment was performed under 2 conditions: once with a dual task (serial subtraction) and once without a dual task, while jumping distance remained consistent across both conditions. ATTd and ITRd were measured using a 3-dimensional motion capture system from 0.25 seconds before initial contact (IC) to 0.75 seconds after IC. Additionally, dynamic knee flexion angle, knee valgus angle, maximal knee flexion moment, and foot angle at IC were measured.

**Results:**

While performing a dual task during the side hop for distance, there was less ATTd during the flight phase and more ITRd during the landing phase (maximal difference [MD], 1.65 mm and 2.07°, respectively; *P* < .001), accompanied by a greater foot angle at IC (MD, 2.71°; *P* < .002), resulting in a more pronounced toe landing. During the triple hop for distance, there was more ATTd and less ITRd around IC while performing a dual task (MD, 1.92 mm and 1.42°, respectively; *P* < .001), accompanied by a decreased knee valgus angle (MD, 0.54°; *P* < .001) and a reduced foot angle (MD, 1.75°; *P* < .05), resulting in a flatter heel landing. No effects of the dual task were observed during the single-leg hop for distance.

**Conclusion:**

Incorporating a cognitive dual task during jump-landing assessments had variable influences on tibiofemoral movements, depending on the assessment. The observed increases in ATTd and ITRd during the landing on certain assessments suggest an increased ACL load and injury risk.

**Clinical Relevance:**

The incorporation of cognitive dual tasks in ACL injury screening and prevention programs should be considered to potentially reduce the risk of injuries.

Sports injuries are becoming increasingly frequent because of the rise in sports exposure.^9^ Among these injuries, anterior cruciate ligament (ACL) injuries are one of the most common noncontact injuries, with over 120,000 injuries occurring annually in the United States,^23,34^ resulting in serious consequences for knee stability and sports participation.^12,27^ An ACL injury often requires ACL reconstruction, especially when aiming to return to sports (RTS).^47^ Despite advancements in surgical techniques and rehabilitation methods,^39^ half of the patients fail to return to their preinjury level of sports,^12^ 1 in 5 athletes reinjures their ACL within 6 years after RTS,^56^ and almost half of the patients develop posttraumatic osteoarthritis.^31^ This emphasizes the importance of identifying risk factors and developing effective ACL injury prevention programs.

Currently, ACL injury prevention programs are primarily based on standardized movements in a predictable environment.^17^ However, recent research highlights the connection between cognitive performance, defined as an athlete’s ability to perform tasks related to working memory, visuospatial capacity and processing speed,^3^ and sport-specific motor skills.^43^ Many sports situations impose high cognitive demands on athletes, especially in sports with open skills (ie, skills affected by the environment), such as predicting the trajectory of the ball or making decisions based on tactical strategies.^43^ As a result, athletes frequently need to allocate their attention across multiple tasks. According to the central capacity sharing model, when 2 simultaneous tasks are performed, limited cognitive recourses are available, resulting in decreased efficacy of one of the tasks.^53^ Therefore, cognitive demands during sports activities can potentially lead to diminished neuromuscular control.^44^

Indeed, in healthy participants, dual tasks have been shown to result in landing mechanics associated with an increased ACL load and (re)injury risk, such as abnormal knee angles, ground-reaction forces, and knee moments.^1,11,32,46^ However, it is not yet known whether dual tasks influence the control of dynamic tibiofemoral movements (ie, anterior tibial translation [ATT] and internal tibial rotation [ITR]). Resisting ATT and ITR is the primary function of the ACL, and inability to control these movements results in increased ACL strain, elevating the risk of (secondary) ACL injuries.^2^ Therefore, this study aimed to investigate the influence of a dual task on dynamic tibiofemoral movements during jump-landing assessments in healthy participants. It was hypothesized that dynamic ATT (ATTd) and dynamic ITR (ITRd) during jump-landing assessments would alter after adding a cognitive mathematical dual task because of a difference in landing mechanics between both conditions. Knee flexion angle, knee valgus angle, maximal knee flexion moment, and foot angle were examined as additional outcomes to evaluate landing mechanics and elucidate potential differences in ATTd and ITRd between conditions.

## Methods

This study was conducted between December 2022 and October 2023. The local ethics committee approved the study design (No. 202200331). All participants were informed about the study protocol and signed informed consent forms before participation.

### Participants

A sample size calculation for repeated-measures analysis of variance (ANOVA) was performed a priori using a correlation between the mean ATTd over time between both legs of *r* = 0.59,^25^ an alpha of .05, an effect size of 0.3, and a power of 0.8 for 1 group and 2 measurements. On the basis of this calculation, 20 healthy participants were included in this study. Inclusion criteria consisted of physically active college students of both sexes who (1) were aged between 18 and 45 years and (2) had no musculoskeletal or neurological impairments. Exclusion criteria consisted of those who (1) had a history of ACL injuries or other severe lower extremity injuries or conditions requiring surgery or that were chronic and (2) were not proficient in the Dutch language to avoid language barriers during explanations.

### Procedure

**Figure 1.**
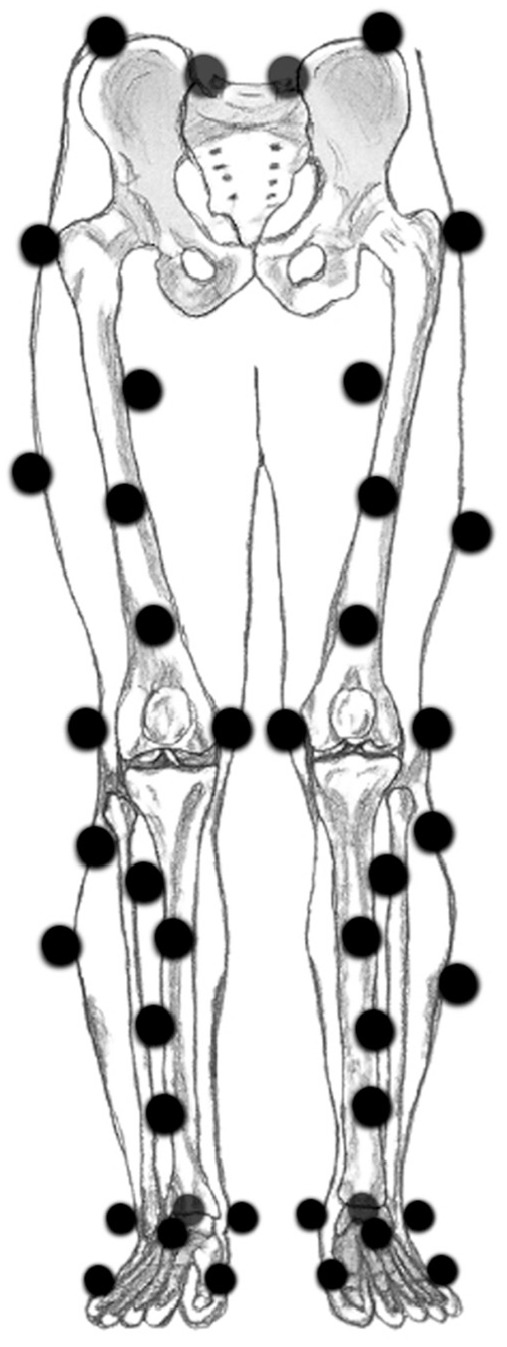
Marker placement. Markers were attached on the left and right posterior superior iliac spine, the left and right iliac crest, the greater trochanter, the medial and lateral epicondyles of the femur, the medial and lateral malleoli of the ankle, the heel, anterior of the talus bone, and the first and fifth metatarsophalangeal joints. An additional 4 markers were placed on the thigh and 6 on the shank.^25^

Each participant was measured in a single session of approximately 2.5 hours. First, a short questionnaire regarding sports activity and injury history was administered. Second, passive ATT was measured with the Lachmeter (Lachmeter Company)^16^ while maintaining a knee flexion angle of approximately 25°. Next, reflective markers for accurately measuring dynamic tibiofemoral movements were placed, as depicted in Figure 1.^24^ The 3-dimensional (3D) marker positions were recorded at a frequency of 200 Hz using a 16-camera 3D motion capture system (Vantage; Vicon).

Calibration data of a star arc movement and of a knee flexion-extension movement were captured (see Quantification of Tibiofemoral Movements for further details). After calibration, participants underwent 3 jumping assessments for both legs: the side hop for distance (SHD), the single-leg hop for distance (SLHD), and the triple hop for distance (THD).^19^ For the SHD, the participants were asked to jump sideways to the same side as the standing leg. For the SLHD, the participants were asked to jump forward, and for the THD, the participants were asked to jump forward 3 times. The following instructions were given: stand on one leg, jump as far as possible, and land on the same leg while maintaining balance for at least 3 seconds without moving the foot. Before formal testing, for each assessment, participants performed 3 practice jumps with both legs to ensure that they understood the instructions. The median distance of these practice jumps served as the starting distance from the center of a 40 × 60–cm force platform (BMS400600HF-OP-2K; AMTI) during testing. Then, all participants performed each assessment 3 times with each leg, both with and without a dual task. The jumping distance toward the force platform remained consistent across both conditions, thereby precluding the evaluation of the dual task’s effect on jumping distance. The order of the 2 conditions was randomized among participants. Additionally, the starting leg was randomized and the same for each (practice) trial during both conditions. The order of assessments was also randomized and the same during the practice and formal jumps as well as during both conditions. All randomizations were performed in MATLAB (Version 9.11; MathWorks) and were included to control for potential training and fatigue effects. Participants wore shorts and gym shoes with the most substantial available grip.

The cognitive dual task was an adapted version of serial subtraction, which is widely used to divide attention across multiple tasks and induces a cognitive load associated with working memory.^11,29^ The investigator named a randomly generated subtraction number between 3 and 9. Then, the investigator named a random number, the starting number, between 20 and 100, which also formed the starting sign for the assessment. The participant commenced the jumping trials while subtracting the subtraction number from the starting number. After 5 to 10 trials, a new randomly generated subtraction number between 3 and 9 was named by the investigator to counter learning effects. The used numbers were randomly generated in MATLAB before testing.

### Outcome Measures

The primary outcome measures were ATTd and ITRd. Secondary outcome measures were dynamic knee flexion angle, knee valgus angle, maximal knee flexion moment, and foot angle at initial contact (IC; defined as the angle between the foot and the ground in the sagittal plane, with positive values indicating a toe landing and negative values indicating a heel landing). These were measured to evaluate landing mechanics, which may provide insight into discrepancies observed in the primary outcome measures. These outcome measures were evaluated in both legs to determine whether the effect of the dual task was similar for both legs (dominant vs nondominant leg). The variables were analyzed during 3 jumping assessments, which are derived from the RTS test battery protocol for patients with ACL injuries.^19^ The selected jumping assessments were the SHD, SLHD, and THD, all performed with a controlled jumping distance determined during the practice jumps. These assessments were chosen because they were developed to evaluate jumping performance, generate high forces around the knee, particularly on the ACL, and involve standardized movements that mimic movements seen in sports activities, highlighting the potential effect of the dual task.^19,50^

### Quantification of Tibiofemoral Movements

For the quantification of ATTd and ITRd, a customized MATLAB script was used based on the method of Boeth et al.^7,25^ To minimize soft tissue artifacts, an optimal rigid body marker configuration was established for the femoral and tibial segments using the optimal common shape technique (OCST).^51^ Subsequently, with both legs, participants performed a star arc movement while standing upright to locate the hip joint centers using the symmetrical center of rotation estimation.^14^ Furthermore, participants performed a knee flexion-extension movement with both legs to determine the local tibiofemoral axes of rotation using the symmetrical axis of rotation approach (SARA).^13^ Utilizing the SARA, 2 local functional axes were reconstructed: one for the femur and one for the tibia in which the motion of each axis was consistent with the motion of its corresponding segment.^13^ Each functional axis was completed with a knee center by projecting the midpoint of the OCST-corrected markers of the medial and lateral epicondyles onto each functional knee axis of rotation. Finally, the ankle center was identified as the midpoint of the 2 OCST-corrected markers of the malleoli.

The femoral coordinate system’s origin was positioned at the knee center of the femoral axis, with its x-axis defined by the normalized femoral axis of rotation determined by the SARA. A temporary z-axis was formed by the normalized vector extending from the fixed knee joint center of the femoral segment to the hip joint center. Similarly, the tibial coordinate system’s origin was positioned at the knee center of the tibial axis, with its x-axis defined by the normalized tibial axis of rotation determined by the SARA. A temporary z-axis was formed by the normalized vector extending from the fixed knee joint center of the tibial segment to the ankle joint center. The y-axes of both coordinate systems were generated by computing the cross-product of their respective x-axis and temporary z-axis. Finally, the coordinate systems were made orthogonal by reconstructing the z-axis using the cross-product of the x-axis and y-axis.

The rotation matrices of the tibial and femoral coordinate systems were determined by transposing their respective coordinate systems. The rotation matrix from the femoral system to the tibial system was computed by dividing the rotation matrix of the tibial system by the rotation matrix of the femoral system. Subsequently, the Euler angles of the femoral coordinate system relative to the tibial coordinate system were computed to determine ITRd, knee flexion angle, and knee valgus angle (Euler sequence: X-Y-Z; flexion/extension–valgus/varus–internal/external rotation).^40^ Then, the distance of the origin of the femoral coordinate system relative to the tibial coordinate system was computed and inverted to determine ATTd. In this manner, tibiofemoral motion is evaluated in the tibial fixed coordinate system so that translation is evaluated in the y-axis of the tibia. The presented method demonstrates high reliability, excellent reproducibility, and high interday repeatability during walking^7^ and high sensitivity during the SLHD.^25^

### Data Analysis

The data were processed and analyzed with MATLAB (Version 9.14). Raw 3D marker position data were filtered using a low-pass frequency convolution filter at 10 Hz with zero lag. Trials with gaps larger than 20 frames were excluded. Gaps smaller than 20 frames were filled using quadratic spline interpolation with zero lag. Force plate data were down-sampled from 2000 to 200 Hz.

ATTd, ITRd, and knee angles were analyzed between 0.25 seconds before IC and 0.75 seconds after IC to include anticipation and feedforward control before the landing as well as the subsequent stabilization phase of the landing itself. IC was determined as the moment when the vertical ground-reaction force on the force plate reached at least 5% of the participant’s body weight. Internal knee flexion moment was derived by standard inverse dynamics calculations,^40^ which were then normalized by the participant’s body weight. The maximal knee flexion moment was identified within the time frame spanning from IC to 0.25 seconds after IC, when the largest forces occur.^36^ Foot angle was analyzed at IC, as this time point influences force absorption of the residual landing,^52^ and was determined with the marker positioned on the first metatarsophalangeal joint and the marker positioned on the heel. Foot angle was computed as the arctangent of the quotient obtained by dividing the difference in height by the distance in the transverse plane of these 2 markers.

The mean ATTd, ITRd, knee flexion angle, knee valgus angle, maximal knee flexion moment, and foot angle were computed based on the mean of the 3 jumps for both legs separately. Trials in which the participant failed to achieve a stationary position within 0.75 seconds after IC were omitted from this computation (<5% of total trials).

### Statistical Analysis

The open-source spm1d package (Version 0.4 [Todd Pataky]; www.spm1d.org) in MATLAB (Version 9.14) was used for statistical analysis of the time-dependent variables: ATTd, ITRd, knee flexion angle, and knee valgus angle. The normality of the data distribution was examined using statistical parametric mapping (SPM) with the Shapiro-Wilk test. If the data followed a normal distribution, SPM with 2-way repeated-measures ANOVA was performed for each jumping assessment to examine potential significant differences between conditions and potential interaction effects between condition and jumping leg. Legs were categorized as the participant’s dominant leg, which they would typically use to kick a ball, and the participant’s nondominant leg. If the data did not follow a normal distribution, statistical nonparametric mapping (SnPM) with 2-way repeated-measures ANOVA with permutations was conducted.^37^ The within-participant factors were condition (dual task or no dual task) and jumping leg (dominant or nondominant), and the dependent variables were ATTd, ITRd, knee flexion angle, and knee valgus angle during the indicated 1 second around IC of the landing. When the SPM test statistic exceeded the critical *F* value based on an alpha <.05, the null hypothesis was rejected, implying a significant difference between conditions.

The Statistics Toolbox of MATLAB (Version 9.14) was used for statistical analysis of maximal knee flexion moment and foot angle at IC. The normality of the data distribution was examined using the Shapiro-Wilk test. If the data followed a normal distribution, 2-way repeated-measures ANOVA was performed for each jumping assessment to examine potential significant differences between conditions. The within-participant factors were condition (dual task or no dual task) and jumping leg (dominant or nondominant), and the dependent variables were maximal knee flexion moment and foot angle at IC. If the data did not follow a normal distribution, the mean of both legs was calculated, and the normality of the data distribution was evaluated again. Depending on the distribution of the data, a paired *t* test or the paired-samples Wilcoxon test was conducted, with condition (dual task or no dual task) as the independent variable and maximal knee flexion moment and foot angle at IC as dependent variables. The significance level for all analyses was set at *P* < .05.

## Results

Baseline characteristics of the participants are displayed in Table 1. None of the participants reported any severe injuries, including concussions, on the questionnaire regarding their injury history.

**Table 1:** Characteristics of Participants (n = 20)

	Mean ± SD or No.
Age, y	22.0 ± 1.7
Sex, male/female	12/8
Height, cm	181.5 ± 8.1
Weight, kg	73.0 ± 10.1
Dominant leg, left/right	3/17
Passive knee laxity,*^*a*^* mm	4.85 ± 1.74
Side hop for distance,*^*a*^* m	1.07 ± 0.27
Single-leg hop for distance,*^*a*^* m	1.63 ± 0.28
Triple hop for distance,*^*a*^* m	4.61 ± 0.65
Pivoting sports,*^*b*^* yes/no	18/2
Sports activity, h/wk	5.89 ± 2.58

a Mean of both legs.

b Defined as sports with regular pivoting and cutting movements.

### Influence of Dual Task on ATTd and ITRd

**Figure 2.**
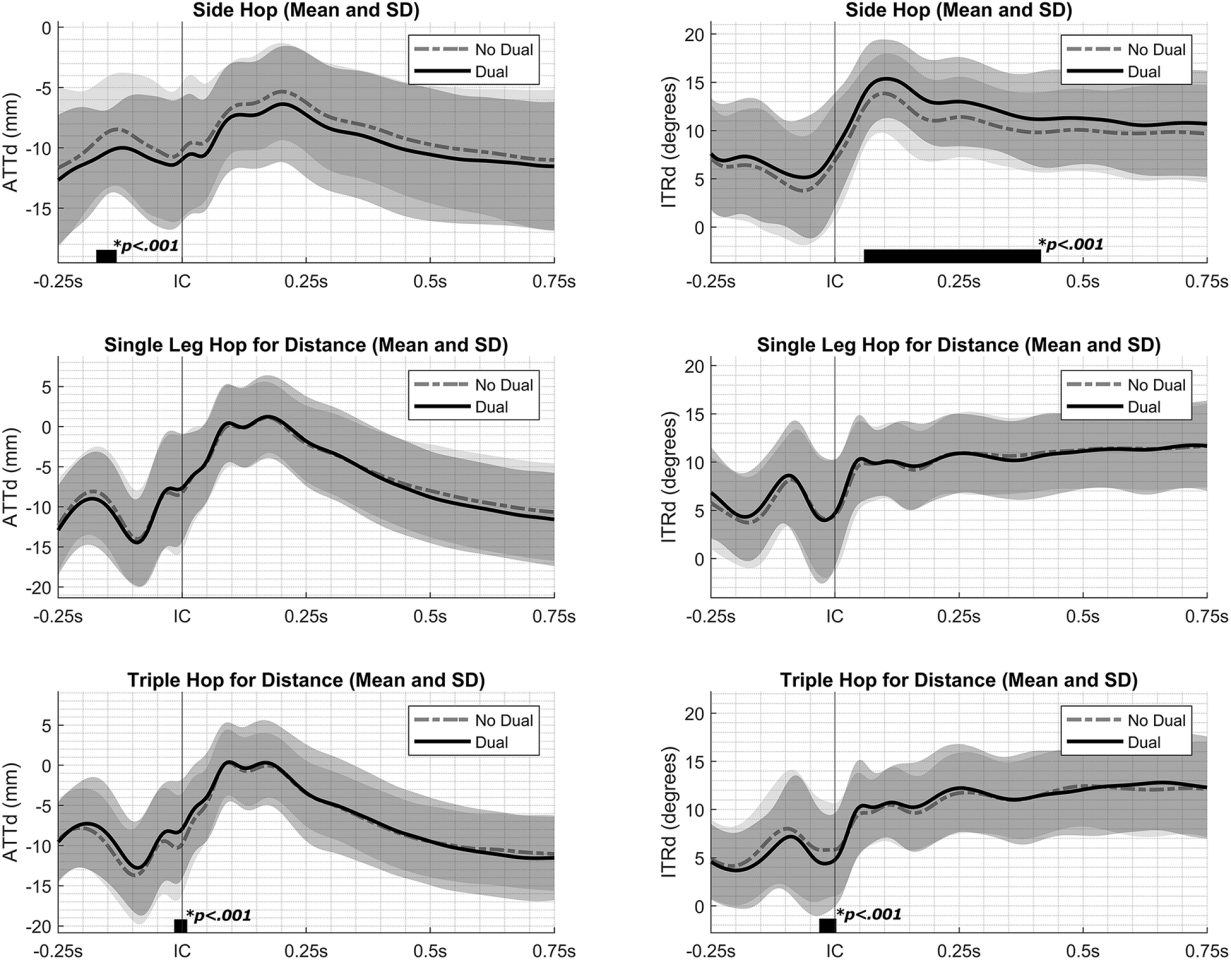
Dynamic anterior tibial translation (ATTd; left) and dynamic internal tibial rotation (ITRd; right) during the side hop for distance, single-leg hop for distance, and triple hop for distance while performing a dual task (solid line) and no dual task (dashed line), including standard deviations (gray areas). An increasing value (less negative or more positive) signified anterior translation and internal rotation of the tibia relative to the femur. Initial contact (IC) is indicated with a vertical gray line. Instances in which the statistical parametric mapping test statistic exceeded the critical threshold are indicated by thick black bars along the x-axis, implying a significant difference between conditions.

During the SHD, participants exhibited less ATTd during the flight phase from 0.17 to 0.13 seconds before IC while performing a dual task compared with no dual task (SnPM(F*) = 9.41; *P* < .001; maximal within-participant difference at 0.14 seconds before IC, 1.65 mm) (Figure 2). Additionally, participants demonstrated more ITRd during the landing phase from 0.06 to 0.42 seconds after IC while performing a dual task compared with no dual task (SPM(F*) = 11.49; *P* < .001; maximal within-participant difference at 0.17 seconds after IC, 2.07°) (Figure 2).

During the THD, participants exhibited more ATTd while performing a dual task compared no dual task from 0.02 seconds before IC to 0.01 seconds after IC (SnPM(F*) = 9.84; *P* < .001; maximal within-participant difference at 0.005 seconds before IC, 1.92 mm) (Figure 2). Furthermore, participants exhibited less ITRd during the THD while performing a dual task compared with no dual task from 0.03 to 0.003 seconds after IC (SnPM(F*) = 10.51; *P* < .001; maximal within-participant difference at 0.02 seconds before IC, 1.42°) (Figure 2).

No significant differences between conditions were observed during the SLHD (*P* > .05) (Figure 2). Similarly, no significant interaction effects between condition and jumping leg were noted across each jumping assessment, indicating that the effect of the dual task was similar for both the dominant and nondominant legs (*P* > .05).

### Influence of Dual Task on Knee Flexion Angle, Knee Valgus Angle, Maximal Knee Flexion Moment, and Foot Angle

During the SHD, participants consistently demonstrated a forefoot landing during both conditions. However, while performing a dual task, participants exhibited a larger foot angle at IC compared with no dual task (*F*(19) = 14.84; *P* < .002; mean, 15.20° vs 12.49°, respectively) (Table 2).

**Table 2:** Maximal Knee Flexion Moment and Foot Angle*^*a*^*

	No Dual Task	Dual Task	Within-Participant Difference	*P*
Side hop for distance				
Maximal knee flexion moment, N·m/kg	2.10 ± 0.53	2.12 ± 0.47	0.02 ± 0.37	.83
Foot angle at IC,*^*b*^* deg	12.49 ± 5.18	15.20 ± 5.48	2.71 ± 4.08	<.002*^*c*^*
Single-leg hop for distance				
Maximal knee flexion moment, N·m/kg	2.76 ± 0.48	2.75 ± 0.49	−0.01 ± 0.30	.87
Foot angle at IC,*^*b*,*d*^* deg	−17.11 (19.87 to −8.32)	−12.47 (−19.77 to −6.20)	0.28 (−0.73 to 1.81)	.22
Triple hop for distance				
Maximal knee flexion moment, N·m/kg	2.94 ± 0.53	2.96 ± 0.62	0.02 ± 0.37	.76
Foot angle at IC,*^*b*^* deg	−16.20 ± 6.91	−14.45 ± 7.31	1.75 ± 3.59	<.05*^*c*^*

a Data are reported as mean ± SD unless otherwise specified. IC initial contact.

b A positive value indicates a forefoot landing, and a negative value indicates a heel landing.

c Statistically significant difference.

d Data are reported as median (interquartile range) because of the nonnormal distribution of data.

**Figure 3.**
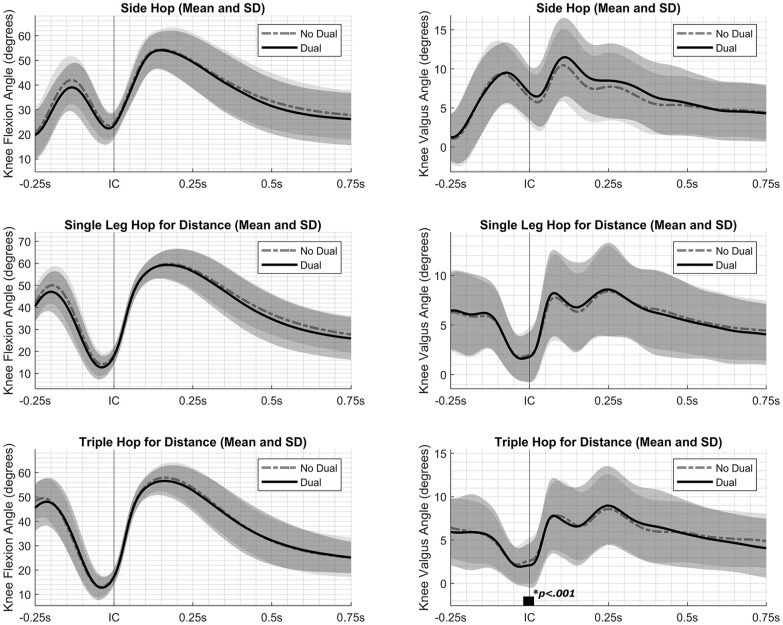
Knee flexion angle (left) and knee valgus angle (right) during the side hop for distance, single-leg hop for distance, and triple hop for distance while performing a dual task (solid line) and no dual task (dashed line), including standard deviations (gray areas). A higher value signified a greater knee flexion angle and knee valgus angle. Initial contact (IC) is indicated with a vertical black line. Instances in which the statistical parametric mapping test statistic exceeded the critical threshold are indicated by thick black bars along the x-axis, implying a significant difference between conditions.

During the THD, participants exhibited a reduced knee valgus angle from 0.02 seconds before IC to 0.01 seconds after IC while performing a dual task compared with no dual task (SnPM(F*) = 9.90; *P* < .001; maximal within-participant difference at 0.01 seconds after IC, 0.54°) (Figure 3). Furthermore, participants consistently demonstrated a heel landing during both conditions. However, while performing a dual task, participants exhibited a smaller foot angle at IC compared with no dual task (*t*(19) = 2.18; *P* < .05; mean, −14.45° vs −16.20°, respectively) (Table 2).

No significant differences between conditions were observed during the SLHD, and no significant differences in knee flexion angle and maximal knee flexion moment were observed across each jumping assessment (*P* > .05) (Figure 3 and Table 2). Similarly, no significant interaction effects between condition and jumping leg were noted across each jumping assessment, indicating that the effect of the dual task was similar for both the dominant and nondominant legs (*P* > .05).

## Discussion

The key findings of this study were that performing the SHD with a dual task compared with the SHD without a dual task resulted in less ATTd during the flight phase and more ITRd during the landing phase as well as a greater foot angle at IC, resulting in a more pronounced toe landing. For the THD, executing a dual task resulted in more ATTd and less ITRd around IC, accompanied by a decreased knee valgus angle and a flatter heel landing. For the SLHD, executing a dual task did not lead to alterations in ATTd or ITRd.

Previous research has demonstrated that dual tasks aimed at inducing a cognitive load can adversely affect motor skills and kinematics, including dynamic stability of the lower extremities and neuromuscular landing mechanics, potentially increasing the risk of ACL (re)injuries.^28,44^ Specifically, recent studies have reported a reduced knee flexion angle at IC, increased maximal knee flexion moment, and larger posterior ground-reaction forces during the simultaneous performance of a jumping assessment and dual task.^11,15,22,41^ These biomechanical alterations suggest heightened quadriceps activation, generating an anterior shear force on the tibia and potentially contributing to increased ATTd.^45^ Moreover, dual tasks are reported to increase the knee valgus angle and moment,^15,57^ contributing to increased ITRd when an anterior shear force is applied on the tibia,^26^ as typically experienced during landing because of quadriceps activation. In contrast to findings of previous studies, the current study did not observe differences in knee flexion angle and maximal knee flexion moment between conditions across all jumping assessments and observed only minimal differences in knee valgus angle. This may be attributed to the difference in jumping assessments between the current and previous studies. Nevertheless, differences in ATTd and ITRd were observed for certain jumping assessments in the current study.

During the flight phase of the SHD, less ATTd was observed while performing a dual task. This could possibly be explained as a result of increased quadriceps-hamstring co-contraction, which reduces the anterior shear force on the tibia compared with dominant agonistic quadriceps activity.^35^ Indeed, previous research has demonstrated increased overall lower extremity muscle activity and quadriceps-hamstring co-contraction during unanticipated cutting maneuvers, occurring even before IC.^4^ During the landing phase of the SHD, more ITRd was observed while performing a dual task. A possible explanation may be an increased knee valgus angle, which is believed to contribute to increased ITRd.^26^ However, despite a trend toward an increased knee valgus angle during the dual task condition in the same time frame, no significant difference was observed between conditions. A potential alternative explanation is the foot-landing technique. Participants exhibited an increased foot angle at IC while performing a dual task during the SHD, resulting in a more pronounced toe landing. During the SHD, a toe landing elicited a ground-reaction force exerted on the forefoot in the medial direction, resulting in inward rotation of the foot and subsequently the tibia. A more pronounced toe landing, as a result of the dual task, would shift the center of pressure anteriorly along the foot, elongating the moment arm and increasing the ITR moment.

During the dual task condition of the THD, participants exhibited more ATTd, less ITRd, a decreased knee valgus angle, and a decreased foot angle, all occurring within a similar time frame around IC. A decreased knee valgus angle contributes to a more vertically aligned femur and tibia, potentially reducing forces in the frontal and transverse planes while increasing forces in the sagittal plane.^26^ Although the difference in knee valgus angle was very small, this could have contributed to the reduced ITRd and the increased ATTd, which occurred within the sagittal plane. The flatter heel landing could be an additional factor that caused more ATTd during the dual task condition. A flatter heel landing limits muscles around the ankle to partly absorb forces,^6^ increasing transmission of the ground-reaction force to the knee.^6,42^ The observed differences in ATTd, ITRd, and knee valgus angle were already apparent slightly before IC, which were likely attributed to distinct landing anticipation mechanisms influenced by the cognitive dual task. This is consistent with the study of Besier et al,^4^ which indicated that the alteration in muscle activation during unanticipated cutting tasks was more pronounced during anticipation of the landing than during the landing itself.

During the SLHD, the lack of disparities seen in knee flexion angle, knee valgus angle, maximal knee flexion moment, and foot angle at IC between conditions corresponded to the absence of variance found in ATTd and ITRd. An explanation for this absence might be that the SLHD is not as cognitively demanding as the THD and SHD. The THD is more demanding because of the requirement for multiple consecutive jumps covering a greater total distance and necessitating the control of larger forces, as evident by the greater maximal knee flexion moment during the landing of the THD compared with the SLHD. In the context of the SHD, knee control is challenged to a greater extent during side hops compared with forward hops in patients with ACL injuries.^33^ During a forward jump, the predominant force is in the sagittal plane, leading participants to engage muscles around the hip, knee, and ankle to control the landing. Conversely, during a sideways jump, participants rely predominantly on hip abductors and adductors to control the landing, presenting a greater challenge. It has been reported that more demanding tasks are more significantly affected by added cognitive loads.^8^

The findings of this study may offer valuable insights into the mechanisms underlying ACL injuries. Video analysis has shown that ACL injuries are most likely to occur during landing or sidestep cutting movements when a player’s attention is directed externally, leading to divided attention between multiple tasks.^54^ The divided attention affects a person’s ability to appropriately detect and respond to a stimulus as well as execute an appropriate motor response, which is thought to arise from competition for limited attentional resources.^21,49^ The present study showed that dividing attention through a cognitive dual task led to certain increases in tibiofemoral movements, such as increased ITRd during the landing of the SHD and increased ATTd around IC of the THD. The primary function of the ACL is to resist ATTd and ITRd,^2^ and reduced control of these movements under external forces can elevate the ACL load and injury risk.^5^ However, it should be noted that the increased ATTd around IC of the THD was accompanied by reduced ITRd, potentially mitigating the increased ACL load. Although the observed alterations in ATTd and ITRd appear to be small, these alterations were identified during controlled landings for standardization purposes. Landing in sports is generally less controlled, requiring more attentional demand and potentially increasing the effect of a dual task. These results may have implications for ACL injury screening and prevention programs. Currently, standard neuromuscular ACL injury prevention programs do not incorporate neurocognitive components associated with an athlete’s ability to sustain motor control while engaging in complex sports environments.^17^ As cognitive dual-task training has been shown to improve landing mechanics,^20^ it is recommended to incorporate these tasks in ACL injury prevention programs to improve athletes’ landing ability under an induced cognitive load to potentially reduce the injury risk. Furthermore, these results might be relevant for RTS test battery protocols for patients with ACL injuries. Current RTS test batteries have been found to be ineffective in reducing the risk of a second ACL injury,^30,55^ and neuroplasticity is considered to be the missing factor in RTS testing.^18^ Patients with ACL injuries show higher activation in primary and premotor areas during simple motor tasks compared with healthy people^38^ and rely more on their visuospatial capacity because of decreased proprioception.^10^ Consequently, athletes with ACL injuries may experience more pronounced inefficacy of a landing when a dual task is added in a sports environment compared with healthy people.^48^ Therefore, the influence of a dual task on tibiofemoral movements may be more pronounced in patients with ACL injuries, emphasizing the need for more research on the effect of dual tasks in these patients and the incorporation of dual tasks into RTS test battery protocols to potentially reduce the reinjury risk.

This is the first study to report the effect of an induced cognitive load on dynamic tibiofemoral movements. However, some limitations of this study need to be addressed. First, while the results of this study are relevant to implications for the primary ACL injury risk, implications for the ACL reinjury risk and RTS test battery protocols remain speculative, as participants had no history of ACL injuries. The second limitation concerns the type of cognitive dual task used, aimed to divide the participant’s attention between multiple tasks and induced cognitive loads associated with working memory. Although this type of cognitive load is involved in sports activities such as strategic planning and decision-making, it does not encompass the entirety of cognitive loads experienced in sports, such as cognitive loads associated with visuospatial capacity, limiting its transferability to the broader sports environment. Lastly, muscle activity was not measured in this study, which could have provided further explanations for the observed differences in tibiofemoral movements between conditions. Therefore, it would be interesting for future studies to examine the influence of a cognitive sport-specific dual task on tibiofemoral movements during jump-landing assessments in patients with ACL injuries while measuring the muscle activity of the lower extremities.

## Conclusion

The addition of a cognitive dual task to jump-landing assessments resulted in variable alterations of tibiofemoral movements, depending on the assessment, potentially related to altered landing mechanics. Several of these alterations, such as the observed increase in ITRd during the landing of the SHD and the increase in ATTd around IC of the THD, are related to an increased ACL load and injury risk, suggesting that divided attention through a cognitive dual task affects a person’s ability to execute an appropriate motor plan during landing. Therefore, the incorporation of cognitive dual tasks in ACL injury screening and prevention programs should be considered to potentially reduce the risk of injuries.
